# Estimated dead space fraction and the ventilatory ratio are associated with mortality in early ARDS

**DOI:** 10.1186/s13613-019-0601-0

**Published:** 2019-11-21

**Authors:** Luis Morales-Quinteros, Marcus J. Schultz, Josep Bringué, Carolyn S. Calfee, Marta Camprubí, Olaf L. Cremer, Janneke Horn, Tom van der Poll, Pratik Sinha, Antonio Artigas, Lieuwe D. Bos, MARS Consortium, Friso M. de Beer, Lieuwe D. Bos, Gerie J. Glas, Janneke Horn, Arie J. Hoogendijk, Roosmarijn T. van Hooijdonk, Mischa A. Huson, Tom van der Poll, Brendon Scicluna, Laura R. Schouten, Marcus J. Schultz, Marleen Straat, Lonneke A. van Vught, Luuk Wieske, Maryse A. Wiewel, Esther Witteveen, Marc J. Bonten, Olaf L. Cremer, Jos F. Frencken, Kirsten van de Groep, Peter M. Klein Klouwenberg, Maria E. Koster-Brouwer, David S. Ong, Meri R. Varkila, Diana M. Verboom

**Affiliations:** 1 Intensive Care Unit, Hospital Universitari Sagrat Cor, Grupo Quironsalud, Carrer de Viladomat, 288, 08029 Barcelona, Spain; 2 Department of Intensive Care, Academic Medical Center, Amsterdam, The Netherlands; 3 Laboratory of Experimental Intensive Care and Anesthesiology, Academic Medical Center, Amsterdam, The Netherlands; 4 Mahidol Oxford Research Unit (MORU), Faculty of Tropical Medicine, Mahidol University, Bangkok, Thailand; 5 Critical Care Center, ParcTaulí Hospital Universitari, Institut d’Investigació i Innovació Parc Taulí I3PT, Universitat Autònoma de Barcelona, Sabadell, Spain; 6 CIBER Enfermedades Respiratorias, Instituto de Salud Carlos III, Madrid, Spain; 7 Department of Medicine, Division of Pulmonary, Critical Care, Allergy and Sleep Medicine, University of California, San Francisco, San Francisco, CA USA; 8 Department of Anesthesia, University of California, San Francisco, San Francisco, CA USA; 9 Cardiovascular Research Institute, University of California, San Francisco, San Francisco, CA USA; 10 Department of Intensive Care Medicine, University Medical Center Utrecht, Utrecht, The Netherlands; 11 Center of Experimental and Molecular Medicine, Academic Medical Center, University of Amsterdam, Amsterdam, The Netherlands; 12 Division of Infectious Diseases, Academic Medical Center, University of Amsterdam, Amsterdam, The Netherlands; 13 Department of Respiratory Medicine, Academic Medical Center, Amsterdam, The Netherlands

**Keywords:** Acute respiratory distress syndrome, ARDS, Respiratory dead space, Dead space, Ventilatory ratio, Intensive care unit, Prognostication, Prediction, Mortality

## Abstract

**Background:**

Indirect indices for measuring impaired ventilation, such as the estimated dead space fraction and the ventilatory ratio, have been shown to be independently associated with an increased risk of mortality. This study aimed to compare various methods for dead space estimation and the ventilatory ratio in patients with acute respiratory distress syndrome (ARDS) and to determine their independent values for predicting death at day 30. The present study is a post hoc analysis of a prospective observational cohort study of ICUs of two tertiary care hospitals in the Netherlands.

**Results:**

Individual patient data from 940 ARDS patients were analyzed. Estimated dead space fraction and the ventilatory ratio at days 1 and 2 were significantly higher among non-survivors (*p* < 0.01). Dead space fraction calculation using the estimate from physiological variables [*V*_D_/*V*_T phys_] and the ventilatory ratio at day 2 showed independent association with mortality at 30 days (odds ratio 1.28 [95% CI 1.02–1.61], *p* < 0.03 and 1.20 [95% CI, 1.01–1.40], *p* < 0.03, respectively); whereas, the Harris–Benedict [*V*_D_/*V*_T HB_] and Penn State [*V*_D_/*V*_T PS_] estimations were not associated with mortality. The predicted validity of the estimated dead space fraction and the ventilatory ratio improved the baseline model based on PEEP, PaO_2_/FiO_2_, driving pressure and compliance of the respiratory system at day 2 (AUROCC 0.72 vs. 0.69, *p* < 0.05).

**Conclusions:**

Estimated methods for dead space calculation and the ventilatory ratio during the early course of ARDS are associated with mortality at day 30 and add statistically significant but limited improvement in the predictive accuracy to indices of oxygenation and respiratory system mechanics at the second day of mechanical ventilation.

## Background

The acute respiratory distress syndrome (ARDS) is an important cause of acute respiratory failure with a high mortality rate [1, 2]. Arterial oxygen tension (PaO_2_) to fraction of inspired oxygen (FiO_2_) is the only measured physiological variable in the Berlin Definition for ARDS [3]. The prognostic value of PaO_2_/FiO_2_ for mortality prediction, however, is limited [4, 5]. More accurate markers are needed to predict outcome in the early course of ARDS.

Both increased V/Q heterogeneity and shunt are the more likely contributors to increased dead space in ARDS. It results from endothelial injury, microvascular plugging with cellular aggregates and thrombi, and disordered pulmonary blood flow, as well as overdistention of alveolar units that may result from the heterogeneity within the injured lung and/or from the use of mechanical ventilation itself [6, 7]. Indeed, dead space fraction (*V*_D_/*V*_T_) during the first week after the initial diagnosis is known to be a predictor of survival, independent of oxygenation [8–10]. Dead space measurements, however, are not utilized routinely in clinical practice, probably due to the added costs of direct measurement techniques. Approximation methods for estimating dead space fraction do not require direct measurement of exhaled carbon dioxide, are less complicated to perform, and feasible to calculate at the bedside [11]. Moreover, estimated dead space correlates well with mortality [12]. Recently, a clinically practical method, the ventilatory ratio (VR), has been validated for estimating pulmonary dead space. It can be calculated using routinely measured respiratory variables at bedside. In patients with ARDS, VR positively correlates with dead space fraction [13], and could, therefore, function as a surrogate for dead space fraction.

In this study, we hypothesized that indices of ventilatory impairment early during the course of ARDS would have independent predictive value for 30-day mortality. In addition, we tested whether estimates of ventilatory impairment at day 2 outperform estimations at day 1.

## Methods

### Study design and ethical considerations

The study design was a post hoc analysis of the ‘Molecular Diagnosis and Risk Stratification of Sepsis’ (MARS) project, a prospective observational cohort study in the mixed medical–surgical intensive care units (ICUs) of two tertiary teaching hospitals in the Netherlands (the Academic Medical Center in Amsterdam, and the University Medical Center in Utrecht) The study was registered at ClinicalTrials.gov (study identifier NCT01905033) [14]. Patients were included via an opt-out method, approved by the medical ethical committees of both hospitals.

### Population

The parent study had the following two inclusion criteria: (a) being admitted to one of the participating ICUs; and (b) having an expected length of stay in ICU longer than 24 h. Patients aged under 18 years were excluded. The current analysis only included patients with ARDS diagnosis during the first 2 days of invasive mechanical ventilation. Patients who were invasively ventilated for less than 24 h were excluded from the analysis.

### ARDS definition

ARDS was defined according to the Berlin Definition [3] as acute hypoxemic respiratory failure, and the concurrent presence of (1) ratio of arterial oxygen tension to inspired fraction of oxygen (PaO_2_/FIO_2_) of 300 mmHg or less; (2) new bilateral pulmonary parenchymal abnormalities on chest X-ray or computed tomography; and (3) ventilatory support with positive end-expiratory pressure (PEEP) of 5 cmH_2_O or more. ARDS was prospectively diagnosed by a team of training research physicians. Case vignettes were used to train the research personnel in identifying chest radiology abnormalities consistent with ARDS.

### Predictors

All formulas used to calculate dead space fraction, including the unadjusted Harris–Benedict [*V*_D_/*V*_T HB_] [15, 16], Penn State [*V*_D_/*V*_T PS_] [16–19], and estimate from physiological variables formulas [*V*_D_/*V*_T phys_] [11, 20], and the VR [21] are provided in Additional file 1. Calculation of the predictors was made once the patient was under mechanical ventilation for more than 24 h and met the ARDS criteria. Arterial blood gas, anthropometrics (height, measured body weight, predicted body weight, body mass index, body surface area, sex and age), and respiratory variables (tidal volume, tidal volume per predicted body weight, respiratory rate, minute ventilation, minute ventilation per predicted body weight, PaCO_2_, PaO_2_/FiO_2_, respiratory system compliance, PEEP, and Murray lung injury score) were recorded. Blood gases were performed every 6 h as part of routine care. For the current analysis, we used the worst values for each day. PaCO_2_ levels and minute ventilation measurements were taken during the same period of the day.

### Endpoints

The primary endpoint was the predictive accuracy of the estimated dead space fraction and VR on mortality at 30 days after ARDS diagnosis measured by area under the receiver operating characteristics curve (AUROCC), net reclassification improvement and integrated discrimination index on top of a model with other predictors of outcome and calibration.

### Analysis plan

All data were analyzed using R studio built under R version 3.2.2 (R Core Team 2013, Vienna, Austria) [22]. Data distribution was assumed not to be normally distributed for descriptive analysis and shown by median and interquartile range. Categorical variables are presented as absolute numbers (percentages). The Kruskal–Wallis test was used to analyze continuous nonparametric data. Categorical data were analyzed by *χ*^2^ tests. *p* < 0.05 was considered to represent a statistically significant difference. Missing data were considered to be missing at random and were imputed using multiple imputation chain equations using the “MICE” package in R [23]. The analyses were repeated with non-imputed data to consider the influence of this step and are reported in Additional file 1: Tables S1 and S2.

Univariate analysis was performed for all respiratory and physiological parameters using logistic regression with 30-day mortality as the dependent variable and was summarized with the odds ratio, 95% confidence interval (95% CI), *p*-value and AUROCC per variable of interest. The independent effect of the estimated ventilation impairment indices was studied by adding the predictor variable to a logistic regression model with five pre-selected variables known to be associated with 30-day mortality: APACHE IV, PEEP, PaO_2_/FiO_2_, driving pressure, and compliance of the respiratory system. The goodness of fit of the logistic regression model was assessed with the Hosmer–Lemeshow test. Variation inflation below 2.5 was considered sufficient to rule out collinearity in the model. The independent association of estimates of impaired ventilation and outcome was visualized by plotting estimated dead space fraction and the covariates against mortality. Estimated dead space fraction and covariates were stratified per tertiles to show trends. Finally, the AUROCC, net reclassification improvement (NRI) and integrated discrimination index (IDI) were used to determine whether estimated ventilation impairment indices improved predictive accuracy on top of that of the base model including PEEP, PaO_2_/FiO_2_, driving pressure and compliance of the respiratory system. All analyses were performed for day 1 and day 2 of ARDS diagnosis. As a secondary analysis, estimated ventilation impairment indexes were compared between Berlin severities of ARDS.

## Results

### Patients

A total of 6994 admissions were included in MARS from January 2011 until December 2013. 965 patients fulfilled the criteria of ARDS, of whom 940 patients were mechanically ventilated for longer than 24 h (Additional file 1: Figure S1). Table 1 shows patient characteristics. Mortality at day 30 was 31%. Non-survivors were older than survivors and had a significantly higher APACHE IV score. Trauma was associated with a lower mortality. Liver cirrhosis and immunodeficiency presented significantly higher mortality.

**Table 1:** Baseline demographics and outcome of 940 patients with acute respiratory distress syndrome

	Survivors *n* = 646	Non survivors *n* = 294	*p*-value
Demographics			
Age (years)	61 (49–71)	63 (53–72)	0.03
Male (%)	399 (62.0)	182 (62.0)	1.00
BMI (kg/m^2^)	25.4 (23–28)	25.2 (22–30)	0.77
APACHE IV score	76 (60–97)	94 (77–122)	< 0.01
Underlying medical illness—no of patients (%)			
Immunodeficiency	111 (17.2)	67 (22.8)	0.05
Chronic respiratory insufficiency	58 (9.0)	30 (10.2)	0.60
Liver cirrhosis	8 (1.2)	11 (3.8)	0.02
Arterial hypertension	201 (31.0)	82 (28.0)	0.31
Diabetes mellitus	101 (15.6)	48 (16.3)	0.83
COPD	75 (11.6)	33 (11.6)	1.00
ARDS etiology—no of patients (%)			
Pneumonia	372 (57.6)	163 (55.4)	0.55
Aspiration	65 (10.0)	29 (10.0)	1.00
Sepsis	385 (59.6)	192 (65.3)	0.08
Pancreatitis	14 (2.4)	8 (2.8)	0.64
Trauma	85 (13.0)	22 (7.5)	0.01
Outcome			
Days of MV	7 (3–16)	5 (2–9)	< 0.01

Data reported as interquartile ratio

*BMI* body mass index, *APACHE* acute physiology and chronic health evaluation, *COPD* chronic obstructive pulmonary disease, *VFD at day 28* ventilator-free days and alive at day 28

### Estimated indices

**Fig. 1:**
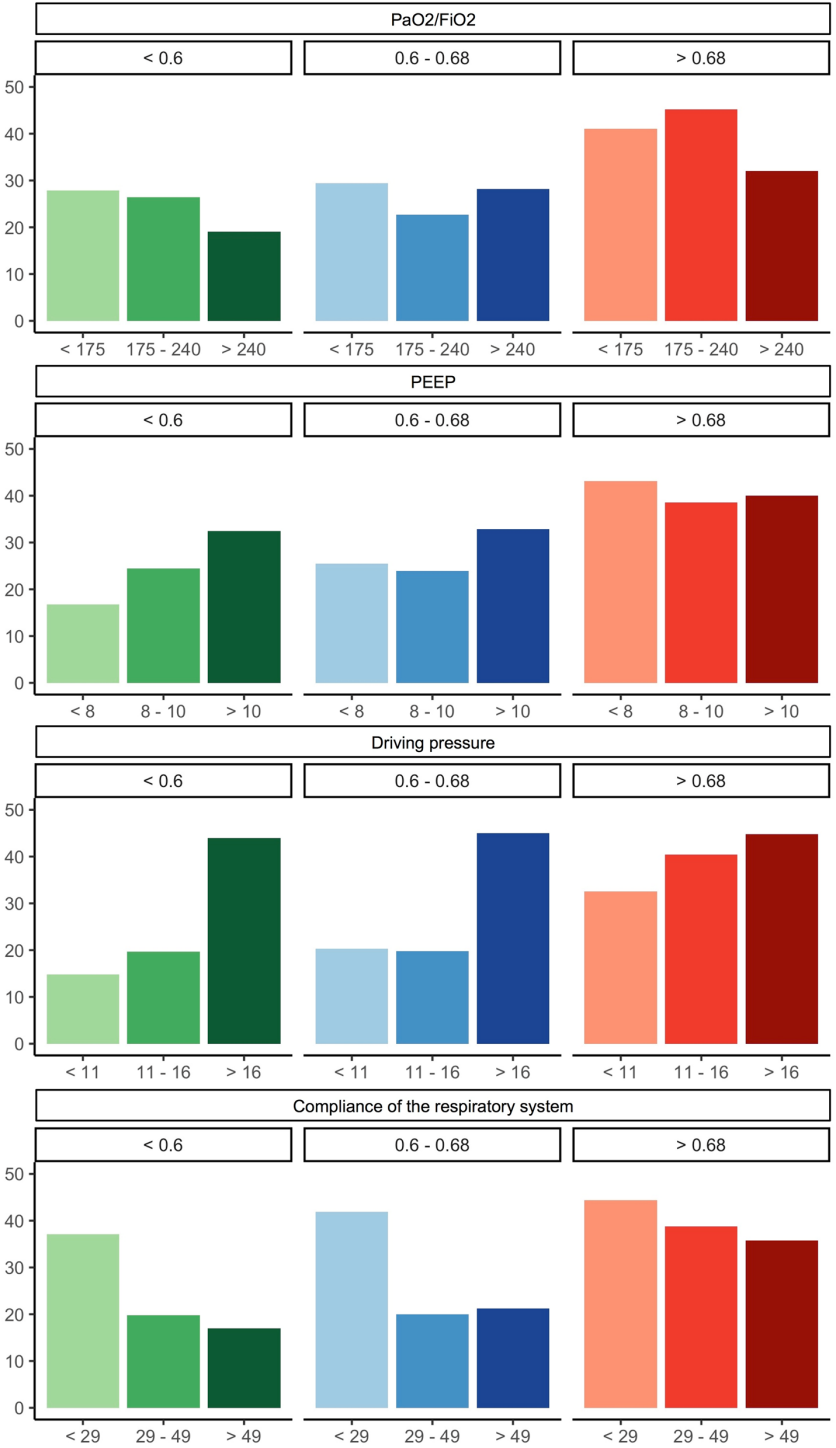
Association between estimated dead space fraction calculated by the Harris-Benedict formula and mortality at 30 days stratified for covariables at day 2 of mechanical ventilation. Estimated dead space fraction (Harris–Benedict estimate) showing the association between *V*_D_/*V*_T_ separated in tertiles (left: < 0.58/middle: 0.58–0.68 and right: > 0.68) and mortality according to tertiles of PaO_2_/FiO_2_ (upper row), PEEP (second row), driving pressure (third row) and compliance of the respiratory system (bottom row)

Driving pressure, respiratory rate, PaO_2_/FiO_2_ and compliance of the respiratory system were significantly different between survivors and non-survivors (Table 2). All the estimated dead space fractions were significantly higher among non-survivors on day 1 and day 2. The VR was also significantly higher among non-survivors on day 1 and day 2 (Table 2). Figure 1 in this manuscript and from Additional file 1: Figure S2 show the mortality per tertile of dead space fraction for the first and second day of after the initial diagnosis, respectively, stratified for tertiles of PaO_2_/FiO_2_, PEEP, driving pressure and compliance of the respiratory system. The figures give a visual representation of the relationship between the estimated dead space fraction and mortality, when stratified for PaO_2_/FiO_2_, PEEP, driving pressure and compliance of the respiratory system. There was no significant difference in estimated ventilation impairment indexes between Berlin severity scores of ARDS on day 1 or day 2 (*p* = 0.49 or higher for each comparison; see Additional file 1: Figures S3 and S4).

**Table 2:** Respiratory variables between survivors and non survivors at days 1 and 2

Variable	Day 1				Day 2			
	Survivors *n* = 652 (QR)	Non survivors *n* = 288 (QR)	OR	*p*-value	Survivors *n* = 638 (QR)	Non survivors *n* = 253 (QR)	OR	*p*-value
PEEP (cmH_2_O)	10 (5–12)	10 (6–13)	1.25 (1.02–1.53)	0.03	8 (5–12)	10 (6–12)	1.48 (1.14–1.92)	< 0.01
Δ*P*	14 (10–19)	17 (11–22)	1.73 (1.39–2.14)	0.04	12 (9–17)	15 (11–21)	2.26 (1.84–2.80)	0.03
PaO_2_/FiO_2_	206 (154–261)	191 (136–255)	0.85 (0.71–1.02)	0.08	214 (160–266)	200 (143–245)	0.76 (0.63–0.92)	< 0.01
Crs (ml/cmH_2_O)	33 (24–50)	27 (20–41)	0.82 (0.72–0.94)	< 0.01	40 (28–66)	30 (20–50)	0.66 (0.56–0.80)	0.01
*V*_D_/*V*_T_								
Harris–Benedict	0.6 (0.6–0.7)	0.7 (0.6–0.7)	1.23 (1.04–1.46)	0.01	0.6 (0.6–0.7)	0.7 (0.6–0.7)	1.25 (1.05–1.50)	0.01
Penn State	0.6 (0.5–0.6)	0.6 (0.5–0.7)	1.28 (1.07–1.55)	< 0.01	0.6 (0.6–0.7)	0.6 (0.5–0.7)	1.40 (1.16–1.70)	0.01
Direct	0.6 (0.6–0.7)	0.7 (0.6–0.8)	1.40 (1.18–1.63)	0.02	0.6 (0.6–0.7)	0.7 (0.6–0.8)	1.56 (1.31–1.85)	0.03
VR	1.6 (1.4–2)	1.8 (1.5–2.3)	1.21 (1.05–1.40)	< 0.01	1.7 (1.4–2)	1.9 (1.5–2.3)	1.30 (1.12–1.51)	< 0.01

*PEEP* positive end-expiratory pressure, *ΔP* driving pressure, *Crs* compliance of the respiratory system, *PaCO*_*2*_ partial pressure of carbon dioxide, *V*_D_/*V*_T_ estimated dead space fraction, *VR* ventilatory ratio

### Mortality prediction with the estimated indices

Each estimate of dead space fraction was significantly associated with mortality at day 30 in unadjusted analyses for day 1 and day 2 (Table 2). After adjustment for predefined covariates (APACHE IV, PEEP, PaO_2_/FiO_2,_ driving pressure and compliance of the respiratory system), the logistic regression model showed independent association with mortality at day 30 for the estimated dead space fraction calculated by the direct formula (*V*_D_/*V*_T phys_) and the ventilatory ratio on day 2 (Table 3). The Hosmer–Lemeshow test indicated a good model fit for each of the tested predictors on days 1 and day 2 (*p*-value = 1). A sensitivity analyses only including patients without any missing data resulted in similar regression coefficients (Additional file 1: Table S1).

**Table 3:** Multivariate analysis of the different *V*_D_/*V*_T_ estimates and VR at days 1 and 2 (*N* = 635)

Variable	Day 1		Day 2	
	OR (95% CI)	*p*-value	OR (95% CI)	*p*-value
*V*_D_/*V*_T_				
Harris–Benedict	1.13 (0.93–1.37)	0.20	1.15 (0.96–1.38)	0.11
Penn State	1.17 (0.96–1.44)	0.11	1.21 (0.98–1.50)	0.06
Direct	1.20 (0.97–1.48)	0.09	1.28 (1.02–1.61)	0.03
VR	1.13 (0.97–1.32)	0.10	1.20 (1.01–1.40)	0.03

Corrected for the following co-variates: APACHE IV, PEEP, PaO_2_/FiO_2_, driving pressure and compliance of the respiratory system

*V*_D_/*V*_T_ estimated dead space fraction, *VR* ventilatory ratio

The AUROCC improved when the estimated dead space calculations and the VR were added to the base model (including APACHE IV score, PEEP, PaO_2_/FiO_2,_ driving pressure and compliance of the respiratory system as predictors) on day 2, but this effect was not observed on day 1 (Table 4). The addition of any estimate of dead space fraction improved the predictive accuracy of the baseline model at day 2 in terms of NRI and IDI but not at day 1 (Table 4).

**Table 4:** Area under the curve (AUC) differences and summary of added value for the predictive variables to the model at days 1 and 2

Variable	With dead space estimation	Base model^a^	*p*-value	NRI (95% IC)	IDI (95% IC)
Day 1					
*V*_D_/*V*_T_					
Harris–Benedict	0.69	0.69	0.68	0.23 (0.09 to 0.36)	0.001 (− 0.001 to 0.004)
Penn State	0.69	0.69	0.82	0.22 (0.09 to 0.36)	0.002 (− 0.001 to 0.005)
Direct	0.69	0.69	0.74	0.15 (− 0.17 to 0.30)	0.002 (− 0.001 to 0.006)
VR	0.69	0.69	0.47	0.10 (− 0.03 to 0.24)	0.002 (− 0.001 to 0.006)
Day 2					
*V*_D_/*V*_T_					
Harris–Benedict	0.72	0.69	< 0.01	0.44 (0.30 to 0.57)	4.36 (2.90 to 5.80)
Penn State	0.72	0.69	< 0.01	0.40 (0.25 to 0.52)	4.46 (3.02 to 5.91)
Direct	0.72	0.69	< 0.01	0.47 (0.33 to 0.60)	4.54 (3.07 to 6.01)
VR	0.72	0.69	< 0.01	0.45 (0.32 to 0.60)	4.51 (3.04 to 6.00)

*V*_D_/*V*_T_ estimated dead space fraction, *VR* ventilatory ratio, *NRI* net reclassification improvement, *IDI* integrated discrimination improvement

^a^Base model includes APACHE IV, PEEP, PaO_2_/FiO_2_, driving pressure and compliance of the respiratory system

## Discussion

The findings of this post hoc analysis of a large cohort of well-defined ARDS patients suggest estimations of impaired ventilation on the second day of ventilation to have an independent association with mortality at 30 days. The estimated additive effects of the predictors are small, statistically significant but with limited improvement and our study did not suggest that one is better than another. Instead, these data suggest that all methods of dead space estimation have similar prognostic value. The added predictive accuracy on top of known predictors of outcome was not observed on the first day of invasive mechanical ventilation. Impaired ventilation does not seem to be associated with the severity of ARDS using the Berlin classification but rather adds an additional dimension to an estimation of the severity of ARDS.

We found that a significant number of ARDS patients had high estimated dead space fractions in the early course of ARDS. These findings confirm those from previous studies that have demonstrated that impaired ventilation can occur within hours after onset of ARDS [8–10, 24]. Dead space is the portion of each tidal volume that does not contribute to CO_2_ clearance and represents a good global index of lung function efficiency. In ARDS, elevated *V*_D_/*V*_T_ results from increased V/Q heterogeneity due to injury of capillaries by thrombotic and inflammatory factors with obstruction of pulmonary blood in the pulmonary circulation and perhaps some degree of overdistention by mechanical ventilation. Shunt and low cardiac output states are known determinants of high *V*_D_/*V*_T_. However, these are features of impaired perfusion rather than ventilation impairment per se [6].

We did not find a statically significant association between PaO_2_/FiO_2_ recorded at the first day of ARDS and the primary outcome. Previous works in ARDS showed that PaO_2_/FiO_2_ may vary due to changes in ventilatory parameters during the first 24 h of mechanical ventilation. These observations may explain why we did not find an association with mortality on the first day in our cohort [24–26].

Our results showed statistically significant but limited improvement in the prediction for mortality for each of the estimated dead space fraction, including the VR. An elevated physiological dead space fraction, calculated with the Bohr–Enghoff equation through volumetric capnography, has been shown to be a strong independent predictor of mortality in the early and intermediate phase of ARDS [8]. The first reports describing the independent association between dead space and mortality in ARDS used a Vt 10 ml/kg of IBW, which is an important factor for increased dead space due to regional pulmonary overdistention or reduced cardiac output [9]. Our findings continue to show an association in mortality among the predictors studied in our cohort with Vt 6 ml/kg IBW, which may suggest intrinsic phenomena on ARDS pathophysiology or maybe other factors different from tidal volume overdistention, such as PEEP.

Dead space fraction was not included in the Berlin Definition. The consensus panel argued that because dead space fraction is challenging to measure, they choose to evaluate minute ventilation standardized at a PaCO_2_ of 40 mmHg as a surrogate instead [3, 27]. Addition of this surrogate resulted in the selection of a smaller group of patients and did not improve mortality stratification. Given the prognostic value of the hereby evaluated surrogates of dead space ventilation, we propose that ARDS patients could be categorized into those with failure of oxygenation and/or those with failure of ventilation. Such categories may facilitate a better understanding between those patients who suffer most from shunt or dead space ventilation. This differentiation could lead to a better understanding of pathophysiological processes and lead to more consistent management strategies. Previous studies have highlighted that the degree of impaired ventilation also depends on the etiology of ARDS [10], e.g., pulmonary ARDS has a significantly higher *V*_D_/*V*_T_ than those with non-pulmonary ARDS. We did not test if there was a difference in the ventilation impairment measurements between pulmonary and extrapulmonary ARDS in our cohort.

The VR has been proposed as an alternative surrogate for dead space fraction. VR, which includes predicted minute ventilation and predicted PaCO_2_, had been described to monitor ventilatory efficiency [28]. Recently, it was found to be independently associated with mortality in ARDS patients [13], and we confirmed this finding. In the current study, VR was independently associated with mortality on day 2, but not on day 1 of ARDS. The advantage of VR is that it is easier to calculate than the estimations of dead space fraction with a similar, but maybe slightly lower predictive accuracy for the outcome.

Important limitations of this study exist. Estimated methods for dead space fraction use equations for energy expenditure or physiological variables [11, 15, 17–19]. The Harris-Benedict estimate predicted the measured dead space fraction with moderate accuracy but was associated with mortality [11]. Recently, the ventilatory ratio has been found to positively correlate with dead space in ARDS [13]. However, the VR has been only validated in mandatory modes of ventilation. It is worth to know that CO_2_ production (V̇CO_2_) changes dramatically once patients are off sedation and in spontaneous modes of ventilation [29]. Both the estimated *V*_D_/*V*_T_ and VR are determined by V̇CO_2_ which is an important factor in CO_2_ homeostasis in critically ill patients and impacts any measure of impaired ventilation [30, 31].

In this study, we did not routinely use controlled ventilation, but spontaneous effort was allowed and likely, due to limited use of sedation. This certainly has led to differences in estimations of dead space fraction as compared to a scenario where all patients would have been under controlled ventilation, and could explain the lack of independent association with mortality in our study, except for the *V*_D_/*V*_T phys_ which not include V̇CO_2_ measurements in its formula. In the future, the evaluation of the estimated measurements of impaired ventilation should incorporate a consideration of altered metabolism as it will impact the variation of CO_2_ production in ICU patients.

It has been described in previous works that the PaO_2_/FiO_2_ values during ARDS may vary and that the values measured during the first 24 h of mechanical ventilation are not as predictive of outcomes [24, 25, 32]. This may be explained by the fact that PaO_2_/FiO_2_ due to some causes can be improved by changing the mechanical ventilation settings, or due to rapid resolution of ARDS, perhaps because the underlying cause has been addressed effectively or because significant fluid overload was treated or prevented. These changes are typically made in the first 24 h leading to rapid improvement in oxygenation in those patients with reversible causes. We believe that this not only applies to oxygenation but also globally all gas exchange derangements, including ventilation impairment. We suspect that impaired ventilation and shunt that can be reversed effectively in the first 24 h does not contribute to mortality and therefore, the association between these parameters and outcome increases after the first day.

However, because we did still confirm the statistically significant but limited improvement in the predictive accuracy of the VR found in other studies, this is actually an advantage of the estimation of impaired ventilation as it increases the external validity of such measurement. Second, we were not able to quantify the correlation between VR and the estimated dead space fraction measurements, due to complexities secondary to mathematical coupling [13]. Third, the association between the studied variables was attenuated after inclusion of the missing data. Because we were aware of this, we reported the lower effect size. On the other hand, the “true” predictive accuracy was statistically significantly higher but with limited improvement after imputation for missing data, as shown by the NRI and IDI, which estimate the added value of the *V*_D_/*V*_T phys_ and the VR for the second day of ARDS on top of the base model. However, although the IDI and the NRI are used to measure and evaluating the improvement in prediction performance of a prognostic marker, we suggest that their results and interpretation should be treated with caution as the interpretation is debated.

Besides these limitations, our study has some strengths. We studied a large prospectively enrolled cohort and, therefore, had sufficient power to detect significant differences in the primary outcome. This was an observational study that included all consecutive patients who developed ARDS in two ICUs over a 3-year period, thereby increasing the generalizability of the findings of the study. Despite using different equations for estimating dead space ventilation fraction, they yielded similar results, suggesting validity of the findings. Furthermore, we used a predefined analysis in line with the latest recommendations for predictive research [33, 34].

The statistically significant but limited improvement of the added predictive value of the variables (dead space estimations and ventilatory ratio) shown in this study indicate that they may be useful bedside indices to monitor impaired ventilation in critically ill patients, and may offer clinicians information about ventilatory failure at the bedside. When direct measurements of dead space fraction are unavailable, dead space estimations may be used in place of direct measurement in clinical practice. Furthermore, the relative simplicity of calculation allows for the incorporation of dead space fraction surrogates in future clinical trials. Given the simplicity of ventilatory ratio to be easily calculated using routine bedside variables, albeit, with known inherent limitations, it may be used as a tool to adjunct mortality estimation in patients with ARDS on day 2.

We believe that rather than a value in itself to be applied in isolation, dead space and its estimates should be understood as a marker to assess ARDS severity and therefore, it could be used to build a model based on oxygenation abnormalities (PaO_2_/FiO_2_) and respiratory mechanics (PEEP) to track respiratory changes to prevent ventilator-induced lung injury or avoid further lung deterioration. Thus, a modified lung injury score may aid in decision-making therapies and risk stratification for future clinical trials.

## Conclusions

Estimations for impaired ventilation on the second day of mechanical ventilation may yield limited but helpful prognostic information that is not captured by conventional oxygenation indices and respiratory system mechanics in patients with ARDS.

## Supplementary information

**Additional file 1.** Estimated dead space fraction (Harris-Benedict estimate) showing the association between VD/VT separated in tertiles (left: < 0.58 / middle: 0.58–0.68 and right: > 0.68) and mortality according to tertiles of PaO2/FiO2 (upper row), PEEP (second row), driving pressure (third row) and compliance of the respiratory system (bottom row).

## Data Availability

All data generated or analyzed during this study are included in this published article and its additional files.

## Abbreviations

APACHE IV: acute physiology and chronic health evaluation IV
ARDS: acute respiratory distress syndrome
AUROCC: area under the receiver operating characteristic curve
FiO_2_: fraction of inspired oxygen
ICU: intensive care unit
IBW: ideal body weight
IDI: integrated discrimination improvement
MARS: molecular diagnosis and risk stratification of sepsis
PaCO_2_: partial pressure of carbon dioxide in arterial blood
PEEP: positive end-expiratory pressure
PaO_2_: partial pressure of oxygen in arterial blood
PaO_2_/FiO_2_: arterial oxygen tension to fraction of inspired oxygen
V̇CO_2_: carbon dioxide production
VR: ventilatory ratio
*V*_D_/*V*_T_: dead space fraction
