# Supplementary material for: Estimated dead space fraction and the ventilatory ratio are associated with mortality in early ARDS
Source: Ann Intensive Care. 2019 Nov 21;9:128. doi: 10.1186/s13613-019-0601-0 (PMC6872683; doi:10.1186/s13613-019-0601-0)
Supplement: Supplementary file 1 — Additional file 1. Estimated dead space fraction (Harris-Benedict estimate) showing the association between VD/VT separated in tertiles (left: < 0.58 / middle: 0.58–0.68 and right: > 0.68) and mortality according to tertiles of PaO2/FiO2 (upper row), PEEP (second row), driving pressure (third row) and compliance of the respiratory system (bottom row). [file 13613_2019_601_MOESM1_ESM.docx]

**Additional file 1**

**Estimated Dead Space Fraction and the Ventilatory Ratio Are Associated with Mortality in Early ARDS**

Luis Morales-Quinteros, MD^1^; Marcus J Schultz, MD, PhD^2,3,4^; Josep Bringué, MsC^5,6^; Carolyn S Calfee, MD, MAS^7,8,9^; Marta Camprubí, PhD^5^; Olaf L Cremer, MD, PhD^10^; Janneke Horn, MD, PhD^2^; Pratik Sinha MD, PhD^7^; Antonio Artigas, MD, PhD^1,5,6^; Lieuwe D Bos MD, PhD^2,3,11^; on behalf of the Molecular Diagnosis and Risk Stratification of Sepsis (MARS) Consortium

^1^Intensive Care Unit, Hospital Universitario Sagrado Corazón, Barcelona, Spain

^2^Department of Intensive Care, Academic Medical Center, Amsterdam, The Netherlands.

^3^Laboratory of Experimental Intensive Care and Anesthesiology, Academic Medical Center, Amsterdam, The Netherlands.

^4^Mahidol Oxford Research Unit (MORU), Faculty of Tropical Medicine, Mahidol University, Bangkok, Thailand.

^5^Critical Care Center, ParcTaulí Hospital Universitari, Institut d’Investigació i Innovació Parc Taulí I3PT, Universitat Autònoma de Barcelona. Sabadell, Spain.

^6^CIBER Enfermedades Respiratorias, Instituto de Salud Carlos III. Madrid, Spain

^7^Department of Medicine, Division of Pulmonary, Critical Care, Allergy and Sleep Medicine; University of California, San Francisco; San Francisco, CA

^8^Department of Anesthesia; University of California, San Francisco; San Francisco, CA

^9^Cardiovascular Research Institute; University of California, San Francisco; San Francisco, CA

^10^Department of Intensive Care Medicine, University Medical Center Utrecht, Utrecht, the Netherlands.

^11^Department of Respiratory Medicine, Academic Medical Center, Amsterdam, The Netherlands.

**Additional methods**

Measurement of the Estimated Physiological Dead-Space Fraction

Estimated dead space fraction for the unadjusted Harris-Benedict [V_D_/V_T HB_], and Penn [V_D_/V_T PS_], state calculation was done by the following formula:

V_D_/V_T_ = 1 – (0.863 x VCO_2_)/(RR x V_T_ x PaCO_2_)

Where VCO_2_ represents CO_2_ production (mL/min), RR is the respiratory rate (breaths/min) and V_T_ is the tidal volume (litres).

VCO_2_ is calculated using the Weir equation^16^:

VCO_2_ = REE/(5.616/RQ) + 1.584

where REE is the Resting Energy Expenditure and RQ is the respiratory quotient, assumed to be 0.8 for this analysis

*Unadjusted Harris-Benedict estimate (REE_HB_)*:

The original sex-specific Harris-Benedict equations were used to estimate the Rest Energy Expenditure (REE)^15^:

Males:

REE_HB_ = 66.473 + 13.752 (Wt) +5.003 (Ht)− 6.755 (age)

Females:

REE_HB_ = 655.096+ 9.563 (Wt) +1.850 (Ht)− 4.676 (age)

*Penn State estimate*

This approach uses the Mifflin-St. Jeor equation (MSJ) to estimate REE for the patient in good health^17^:

Males:

REE_MSJ_ = 10 (Wt) + 6.25 (Ht) – 5 (age) + 5

Females:

REE_MSJ_ = 10 (Wt) + 6.25 (Ht) – 5 (age) – 161

with weight (Wt) in kg, height (Ht) in cm, and age in years.

Additional clinical variables are then incorporated to yield the Penn State equations (PSU) for REE in critical illness^18,19^:

If BMI < 30 kg/m^2^:

REE_PSU_ = 0.96 (REE_MSJ_) + 31 (RR) (V_T_) + 167(T*_max_*) – 6212

If BMI ≥ 30 kg/m^2^:

REE_PSU_ = 0.71 (REE_MSJ_) + 64 (RR) (V_T_) + 85(T*_max_*) – 3085

where BMI is the body mass index, RR is the respiratory rate (breaths/min), V_T_ is the tidal volume (liters), and T*_max_* is the maximum temperature (°C) over the last 24 hours.

*Estimate from physiological variables* [V_D_/V_T phys_]^11^:

V_D_/V_T_ = 0.1726 + 0.0059 (RR) + 0.0054 (PEEP) + 0.0293 (LIS) + 0.0036 (PaCO_2_ x VE) + 0.000057 (PaCO_2_ x age)

where RR is the respiratory rate (breaths/min), PEEP represents set PEEP (cm H_2_O) on the mechanical ventilator, LIS is the Murray lung injury score. PaCO_2_ is measured in mmHg, and VE represents total minute ventilation (L/min).

Measurement of the Ventilatory Ratio (VR)^21^

VE*_measured_* x PaCO_2_ *_measured_*

VR = VE*_predicted_* x PaCO_2_ *_ideal_*

Where VE*_measured_* is the measured minute ventilation (mL/min), PaCO_2_ *_measured_* is the measured arterial pressure of carbon dioxide (mmHg), VE*_predicted_* is the predicted minute ventilation calculated as predicted body weight X 100 (mL/min), and PaCO_2_ *_ideal_* is the expected arterial pressure of carbon dioxide in normal lungs if ventilated with the predicted minute ventilation. PaCO_2_ *_ideal_* is set as 37.5 mmHg for all patients.

**Table S1. MULTIVARIATE ANALYSIS OF THE DIFFERENT V_D_/V_T_ ESTIMATES AND VR AT DAYS 1 AND 2* FOR PATIENTS WITHOUT ANY MISSING DATA (N = 635).**

| VARIABLE | DAY 1 | | DAY 2 | |
| --- | --- | --- | --- | --- |
|  | **OR (95%CI)** | **P-value** | **OR (95%CI)** | **P-value** |
| Vd/Vt |  |  |  |  |
| Harris-Benedict | 1.37 (1.07-1.75) | <0.01 | 1.48 (1.09-2.00) | 0.01 |
| Penn State | 1.36 (1.05-1.76) | 0.02 | 1.43 (1.03-1.97) | 0.02 |
| Direct | 1.48 (1.11-1.98) | <0.01 | 1.43 (1.03-1.97) | 0.01 |
| VR | 1.28 (1.04-1.56) | 0.01 | 1.23 (0.99-1.54) | 0.06 |

*corrected for the following co-variates: APACHE IV, PEEP, PaO_2_/FiO_2_, driving pressure and compliance of the respiratory system. V_D_/V_T_, estimated dead space fraction; VR, ventilatory ratio

**Table S2. NUMBER OF MISSING RESPIRATORY VARIABLES AT DAY 1 AND DAY 2**

| VARIABLE | DAY 1 | DAY 2 |
| --- | --- | --- |
| Vt/PBW (ml/kg/PBW) | 137 | 194 |
| RR (breaths/min) | 55 | 96 |
| PEEP (cmH_2_O) | 141 | 193 |
| ΔP | 142 | 193 |
| Crs (ml/cmH_2_O) | 144 | 195 |
| PaCO_2_ (mmHg) | 107 | 149 |
| LIS | 208 | 250 |
| Vd/Vt |  |  |
| Harris-Benedict | 0 | 0 |
| Penn State | 0 | 0 |

Vt, tidal volume adjusted for predicted body weight; RR, respiratory rate; PEEP, positive end-expiratory pressure; ΔP, driving pressure; Crs, compliance of the respiratory system; PaCO_2_, partial pressure of carbon dioxide; LIS, Lung Injury Score; V_D_/V_T_, estimated dead space fraction

**Additional file FIGURE TITLES AND LEGENDS**

**Figure S1. FLOW CHART OF THE INCLUDED PATIENTS**

**
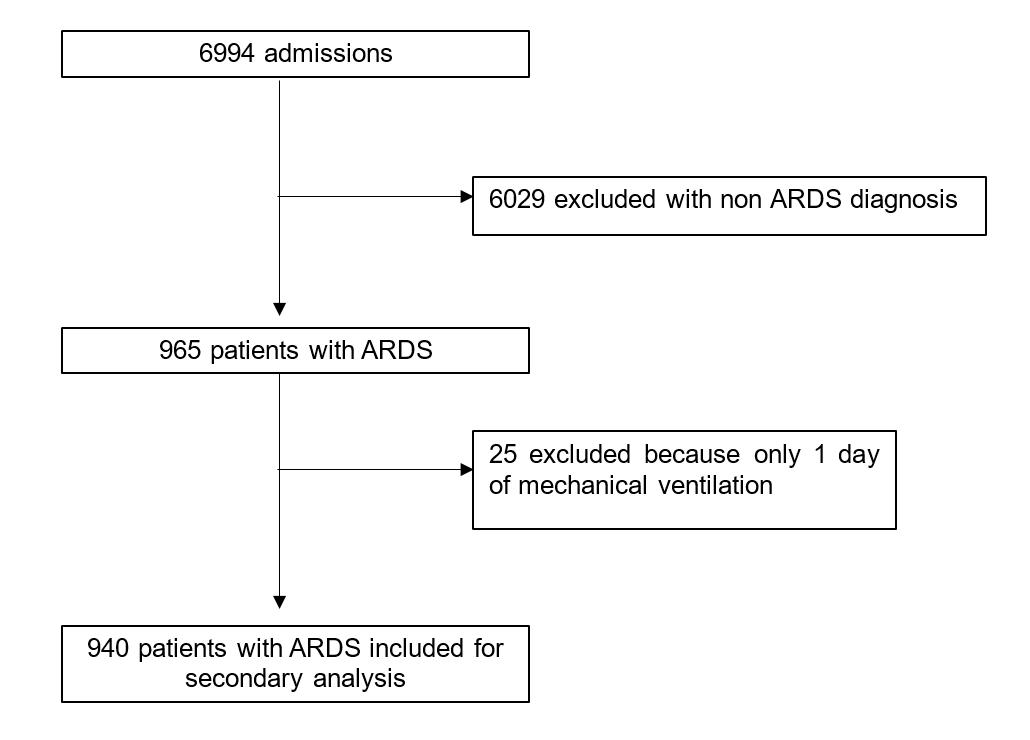
**

**FIGURE S2. ASSOCIATION BETWEEN ESTIMATED DEAD SPACE FRACTION CALCULATED BY THE HARRIS-BENEDICT FORMULA AND MORTALITY AT 30 DAYS STRATIFIED FOR COVARIABLES AT DAY 1 OF MECHANICAL VENTILATION**

Estimated dead space fraction (HB estimate) showing the association between V_D_/V_T_ separated in tertiles (left: <0.58 / middle: 0.58-0.68 and right: >0.68) and increase mortality independent of (upper row) PaO_2_/FiO_2_ (second row) PEEP (third row) driving pressure and (lower row) compliance of the respiratory system stratified in tertiles.

**
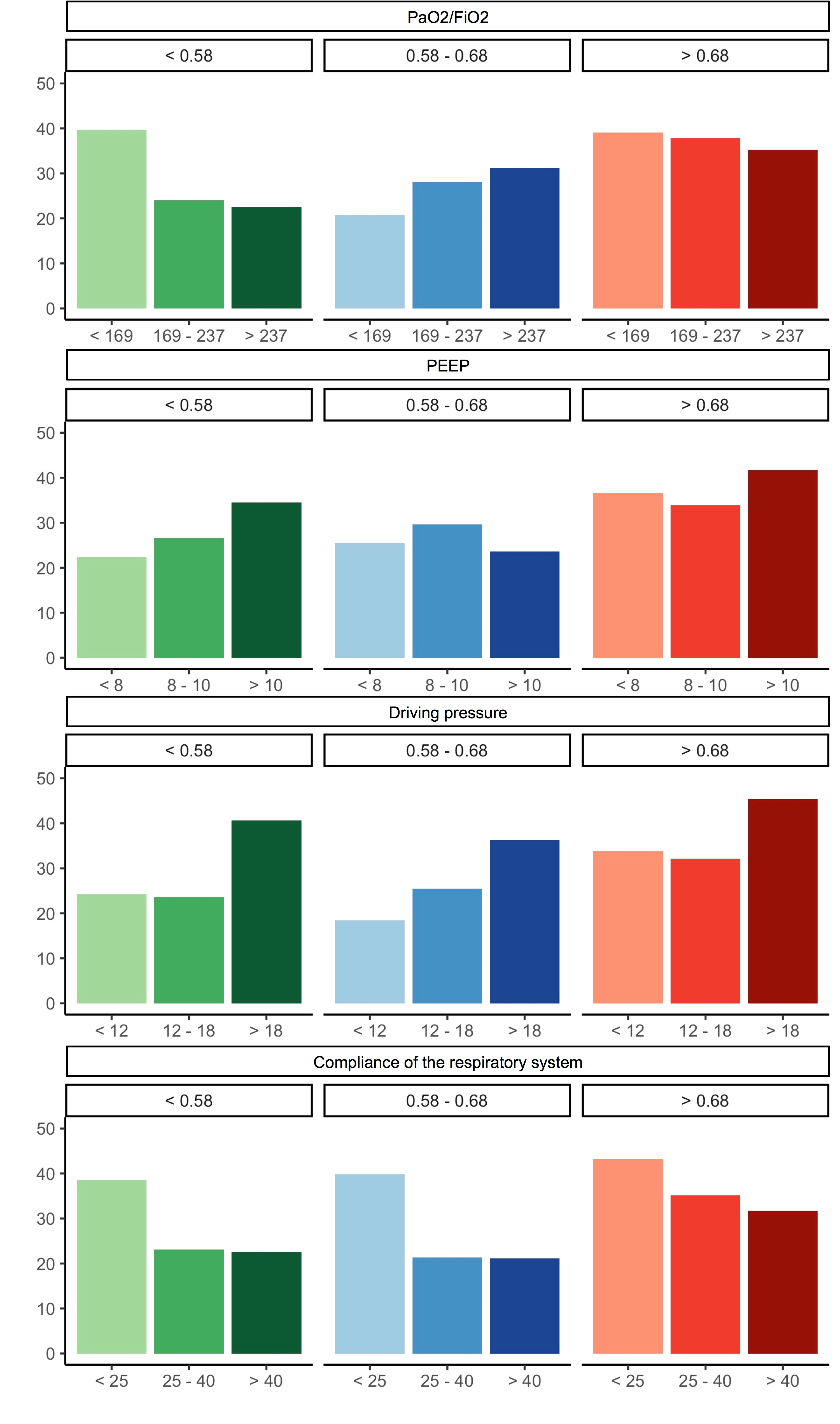
**

**FIGURE S3. ESTIMATED DEAD SPACE FRACTION MEASUREMENTS STRATIFIED BY ARDS SEVERITY AT DAYS 1 AND 2**

ARDS, acute respiratory distress syndrome; HB, Harris-Benedict; PS, Penn State

**
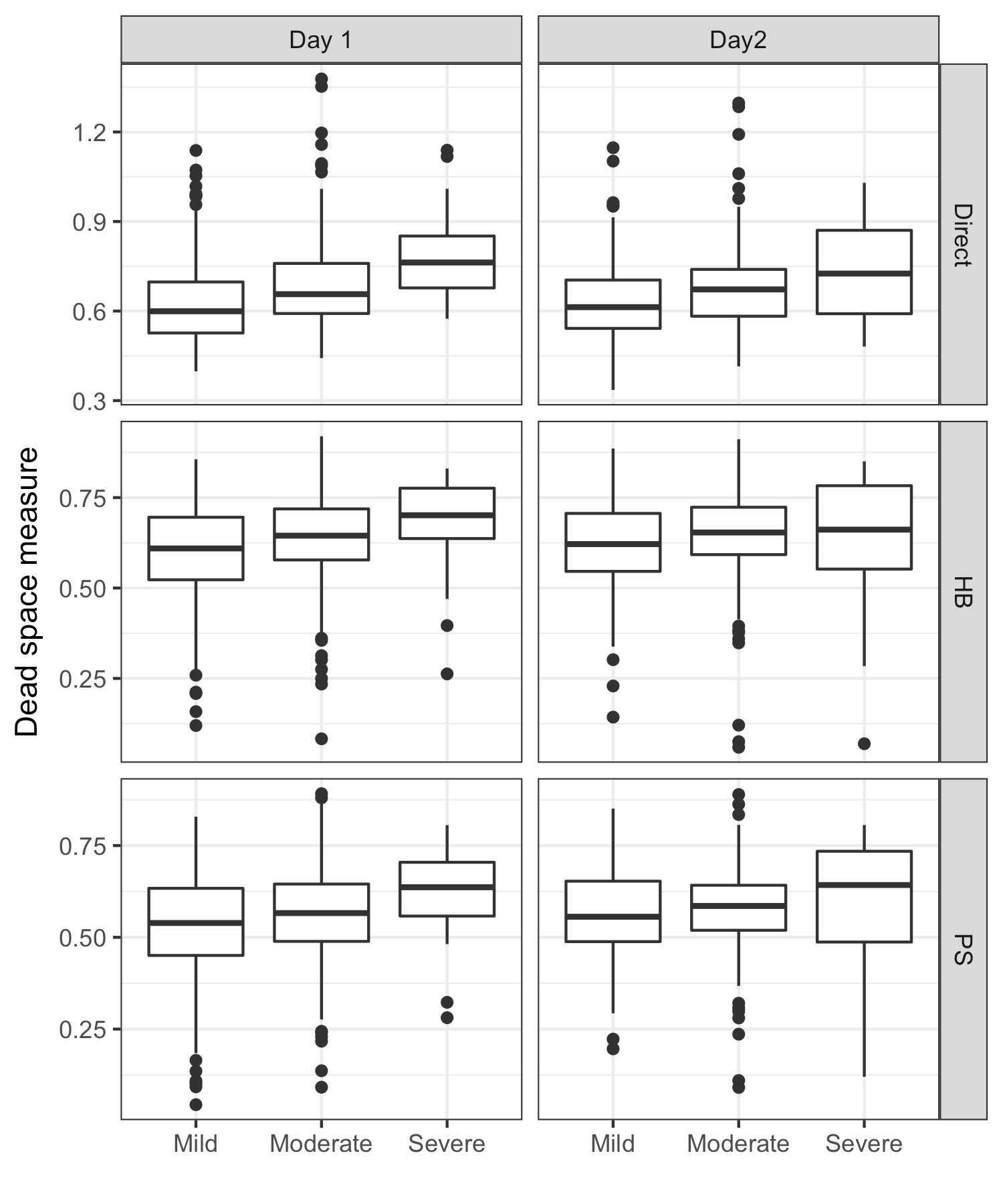
**

**FIGURE S4. VENTILATORY RATIO MEASUREMENTS STRATIFIED BY ARDS SEVERITY AT DAYS 1 AND 2.**


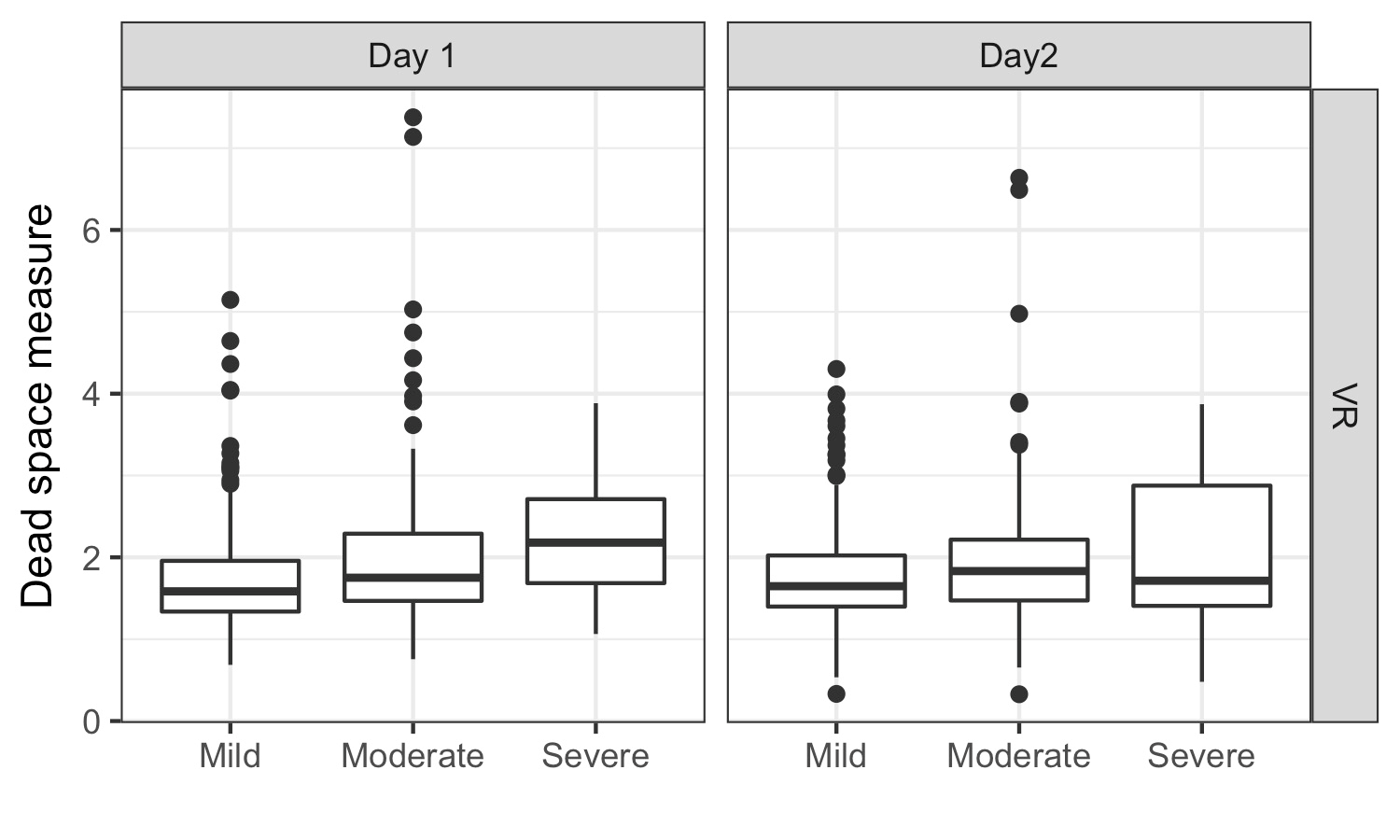


ARDS, acute respiratory distress syndrome; VR, ventilatory ratio
